# Supplementary material for: Characterization of platinum(II) complexes exhibiting inhibitory activity against the 20S proteasome
Source: R Soc Open Sci. 2020 Aug 19;7(8):200545. doi: 10.1098/rsos.200545 (PMC7481701; doi:10.1098/rsos.200545)
Supplement: ff-target activity of Pt complexes, inhibitory activities of noegative cotrol against proteasomes and chemical structure of N-acetyl cysteine adduct [file rsos200545supp1.docx]

Electronic supplementary material

Characterization of platinum(II) complexes exhibiting inhibitory activity against 20S proteasome

Tatsuto Kiwada^1^, Hiromu Katakasu^2^, Serina Okumura^3^, and Akira Odani^1^

^1^Faculty of Pharmacy, Institute of Medical, Pharmaceutical, and Health Sciences, Kanazawa University, Kakuma-machi, Kanazawa, 920-1192, Japan

^2^School of Pharmaceutical Sciences, College of Medical, Pharmaceutical and Health Sciences, Kanazawa University,

Kakuma-machi, Kanazawa, 920-1192, Japan

^3^School of Pharmacy, College of Medical, Pharmaceutical and Health Sciences, Kanazawa University,

Kakuma-machi, Kanazawa, 920-1192, Japan

Corresponding author: Tatsuto Kiwada (kiwada@p.kanazawa-u.ac.jp), Akira Odani (odani@p.kanazawa-u.ac.jp)

Off-target activity against other proteases

Inhibition of cathepsin B

Cathepsin B (50 ng) was incubated in 100 μL assay buffer (100 mM sodium actetate, 5 mM EDTA, 5mM DTT; pH 5.5) with different concentrations of platinum complexes and 20 μM of the fluorogenic peptide substrate Z-RR-MCA for 1 h at 37°C. After incubation, the production of hydrolyzed AMC groups was measured at 450 nm (λ_ex_ = 365 nm).

Inhibition of α-chymotrypsin

**
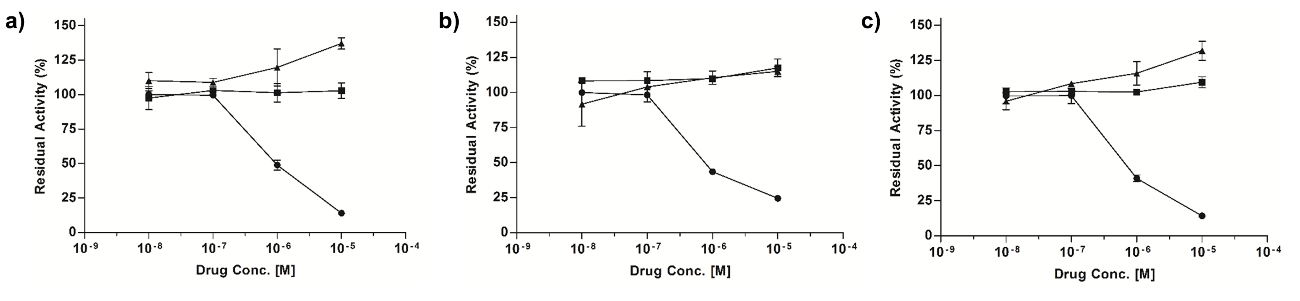
**In 100 μL assay buffer (25 mM HEPES and 0.5 mM EDTA, pH 7.8), α-chymotrypsin (50 ng) was incubated with different concentrations of platinum complexes and 25 μM of the fluorogenic peptide substrate Suc-LLVA-MCA for 1 h at 37°C. After incubation, the production of hydrolyzed AMC groups was measured at 450 nm (λex = 365 nm).

**Figure S1**. Residual activity of cathepsin B (■), α-chymotrypsin (▲) and 20S proteasome (●) in the presence of complex **1**(a), complex **2** (b) and complex **3** (c).

**Inhibitory activity of N-9-anthracenylmethyl-1,2-ethanediamine and (ethylenediamine)bispyridine platinum(II) chloride against the purified 20S proteasome**


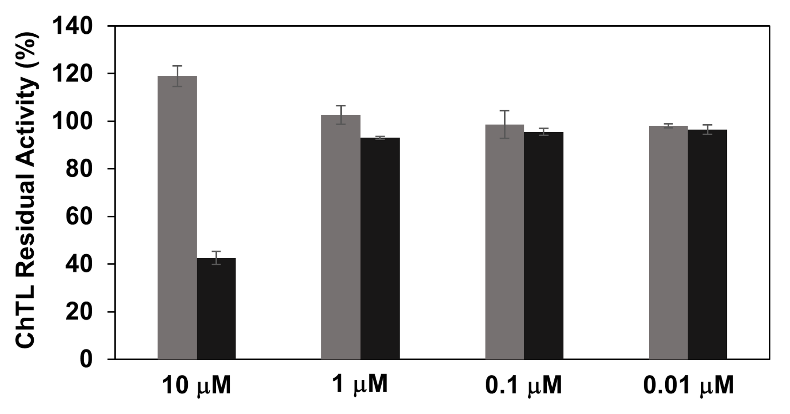
For the proteasome inhibition assays, the peptide-AMC substrates (25 μM Suc-LLVY-AMC) and inhibitors were added to assay solutions. The assay buffer had the following composition: 25 mM HEPES, 0.5 mM EDTA, 0.03% SDS (pH 8.0). Human erythrocyte 20S proteasome (100 ng) was added to the assay buffer containing substrates and inhibitors at a final volume of 100 μL. After incubation at 37°C for 1 h, the fluorescence emission spectrum at 450 nm (λex, 365 nm) was measured by using a fluorescence microplate reader.

**Figure S2**. Inhibitory rate of (ethylenediamine)bispyridine platinum (II) chloride (gray) and N-9-anthracenylmethyl-1,2-ethanediamine (black) against ChTL activity of the purified 20S proteasome.

**Inhibitory activity of N-9-anthracenylmethyl-1,2-ethanediamine against the 20S proteasome in the whole cell lysate**

For the determination of inhibitory activity in the whole cell lysate, peptide-AMC substrates (50 μM Suc-LLVY-AMC), inhibitors, and the whole cell lysate (15 μg) were added to the assay solutions. The following assay buffer was used: 25 mM HEPES, 0.5 mM EDTA, 0.03% SDS (pH 8.0). After incubation at 37 °C for 4 h, the fluorescence emission was measured at 450 nm (λex = 365 nm) by using a fluorescence microplate reader.

**
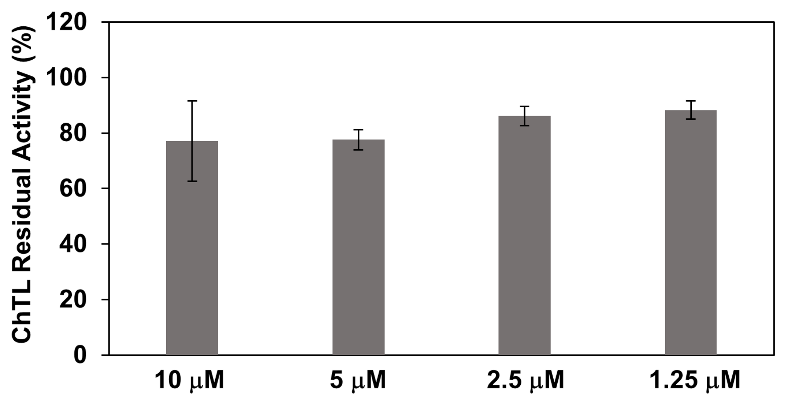
**

**Figure S3**. Inhibitory rate of N-9-anthracenylmethyl-1,2-ethanediamine against ChTL activity of the 20S proteasome in the whole cell lysate.


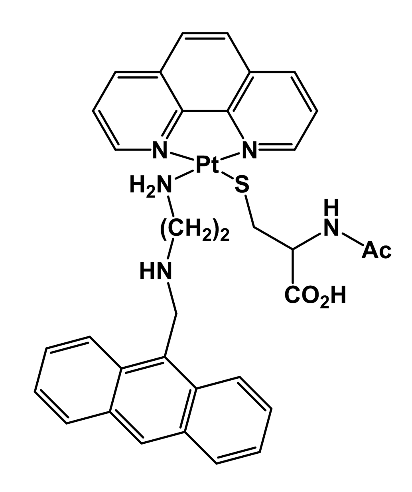


**Figure S4.** The plausible structure of 1:1 adduct of complex **2** with N-acetylcysteine.
